# Seamless Tracing of Human Behavior Using Complementary Wearable and House-Embedded Sensors

**DOI:** 10.3390/s140507831

**Published:** 2014-04-29

**Authors:** Piotr Augustyniak, Magdalena Smoleń, Zbigniew Mikrut, Eliasz Kańtoch

**Affiliations:** AGH-University of Science and Technology, 30, Mickiewicz Ave., 30-059 Kraków,Poland; E-Mails: msmolen@agh.edu.pl (M.S.); zibi@agh.edu.pl (Z.M.); kantoch@agh.edu.pl (E.K.)

**Keywords:** ambient assisted living, surveillance, home care, aging society

## Abstract

This paper presents a multimodal system for seamless surveillance of elderly people in their living environment. The system uses simultaneously a wearable sensor network for each individual and premise-embedded sensors specific for each environment. The paper demonstrates the benefits of using complementary information from two types of mobility sensors: visual flow-based image analysis and an accelerometer-based wearable network. The paper provides results for indoor recognition of several elementary poses and outdoor recognition of complex movements. Instead of complete system description, particular attention was drawn to a polar histogram-based method of visual pose recognition, complementary use and synchronization of the data from wearable and premise-embedded networks and an automatic danger detection algorithm driven by two premise- and subject-related databases. The novelty of our approach also consists in feeding the databases with real-life recordings from the subject, and in using the dynamic time-warping algorithm for measurements of distance between actions represented as elementary poses in behavioral records. The main results of testing our method include: 95.5% accuracy of elementary pose recognition by the video system, 96.7% accuracy of elementary pose recognition by the accelerometer-based system, 98.9% accuracy of elementary pose recognition by the combined accelerometer and video-based system, and 80% accuracy of complex outdoor activity recognition by the accelerometer-based wearable system.

## 1. Introduction

Maintaining the independent life and professional activity of the elderly is subjectively perceived as improvement of life quality, and is also in the interest of societies which benefit from their experience and wisdom. Combining mature techniques of medical diagnostics with traditional surveillance system solutions leads to a generalized approach to health- and activity-based supervision of elderly, providing the comfort of independence together with security of seamless monitoring and alerting.

Thanks to its potential impact on the wellbeing in aging societies, the topic is currently considered among most essential and thus generously funded and intensively investigated on both the system and sensor levels. Various sensors proposed recently follow home-care-oriented design guidelines: small size, light weight, unobtrusive operation, long autonomy, *etc*. These sensors are usually developed for a specific disease-related purpose, and following the demand of most frequent age-related disorders. Early applications were developed mainly for diabetic [1,2], asthmatic [3,4] or cardiac [5,6] patients, but recently remote monitoring also includes gait analysis [7,8] and fall detection [9,10]. Accordingly to the paradigm of minimum obtrusive measurements, wearable sensors rarely resemble a traditional Holter recorder, but are built in form of bracelets, chest belts [11] or sensorized clothes [12].

A separate line of research follows the achievements of digital telecommunication technologies such as wireless networks and has yielded several solutions for architecture and data flow in body sensor networks (BSN) [13–15]. An exhaustive review of these solutions may be found in [16] and [17]. A complementary approach originates from the idea of smart home (or smart environment) which initially was focused on pleasure or commodity, but currently stresses more the provision of healthcare services [18–20]. Such environments usually apply touchless visual (or infrared) detection of presence [21,22], radar [23] or ultrasound [24] measurement of movement. An interesting, fast developed domain are sensors embedded in common home appliances (such as beds or bathtubs) recording their operation by the monitored person [25,26].

A thorough revision of available scientific papers and commercial products led to formulating a list of features, requisite in current telemedicine systems for seamless tracing of human behavior and health status. These features include:

- usage of open sensor network architectures instead of focus on a particular disease
- design for multipurpose health prevention instead of for follow-up of patients with known medical records
- real-time cooperation of premise-embedded and wearable sensor networks
- consideration of the habits of the monitored person and the expected usage of the environment in qualifying the behavior
- lack of territorial restrictions allowing for seamless recording of behavior with unconstrained mobility of the monitored person

We are striving for a design of a backbone surveillance infrastructure composed of premise-embedded, area-related sensors cooperating with wearable, subject-specific sensors conditionally networked and supervised by an independent server, according to the habits of each particular individual and expected usage of the environment. The growing demand for such solutions, monitoring human behavior and health status, is justified by longer life expectancy, demographic changes and looser familiar relations observed in developed countries.

This paper is organized as follows: Section 2 presents several recent works on selected aspects concerned in our system design. Section 3 presents a general overview of the system architecture, while Section 4 describes selected original ideas of the methodology which includes: management of data and hardware resources, detection of presence and pose, complementary measurements for recognition of activity and learning of behavior and detecting of danger. In Section 5 we describe the details of the experiments and their results. Finally, in Section 6 conclusions and future work are presented.

## 2. Related Works

Responding to the need for elderly-dedicated environments, many researchers currently focus on assisted living solutions and the development of monitoring systems as wearable or embedded in smart homes infrastructures. The primary goal of such systems is detection of potentially dangerous events (e.g., fall detectors [27]) and recognition of human pose or action [28]. More sophisticated systems identify and classify activities of daily living (ADL) and learn the habits of supervised subjects [29]. Various methodologies were already proposed for sensor set, sensor data processing, behavior recognition and classification.

Among a vast number of papers concerning assisted living solutions, we have to mention first a comprehensive review of ambient-assisted living tools for older adults, recently published by Rashidi *et al.* [30]. An interoperability and quality-oriented study of ambient assisted living frameworks by Memon *et al.* [31] investigated the critical issues from the design, technology, quality-of-service, and user experience perspectives, and Damas *et al.* [32] proposed a system architecture based on the Open Services Gateway Initiative, which offers plug-and-play connectivity of ambient assisted living devices. Matern *et al.* [33] proposed the use of conditional random fields that allow for adding and removing sensors in an easy and efficient way. Aquino-Santos *et al.* [34] identified arrhythmias as a typical age-related disease and included arrhythmia monitoring in the system architecture.

Several interesting methods for recognition of human activity were investigated and reported as not dependent on the surveillance system used. In one of the earliest papers Lühr *et al.* [35] proposed a data mining approach to distill the behavior from patterns from sensor event logs. The paper by Hong *et al.* [36] addressed the fusion process of contextual information derived from uncertain sensor data. In [37] a Zernike moments-based unified framework for human behavior recognition was proposed. Ros *et al.* [38] proposed a solution of behavior recognition problem based on learning automata and fuzzy temporal windows. The system learns the normal behaviors, and uses that knowledge to distinguish normal and abnormal human activities in real time.

With the production of ultra low power acceleration sensors, these devices become widely used for wearable health and medical monitoring applications. An interesting survey of mobile classification algorithms for accelerometer-based activity recognition was presented by Ayu *et al.* [39]. Garcia-Ceja *et al.* [40] presented an accelerometer-based wearable system performing a long-term activity recognition based on distribution of simple activities represented as a histogram. Amini *et al.* [41] reported on using accelerometers to capture motion data to estimate the location of the device on the user's body, using mixed supervised and unsupervised time series analysis methods. The papers of Bagala *et al.* [42] and Bourke *et al.* [43] are focused on fall detection, but also lie within the scope of this paper, since fall detection is one of the primary aspects of safety at home and outdoors. The paper by Liu *et al.* [44], although not directly related to the elderly, demonstrated the use of Dynamic Time Warping (DTW) in gesture recognition based on accelerometer signals.

Among sensors embedded in the smart home infrastructure, the most human-like and most widely used sensing methods are based on image analysis. An exhaustive review of vision-based human action recognition was presented by Poppe [45]. Another survey of vision-based methods for action representation, segmentation and recognition was presented by Weinland *et al.* [46]. The paper by Chen [47] provided an interesting survey of the use of depth imagery (e.g., Microsoft Kinect) for analyzing human activity. Li *et al.* [48] presented a template-matching algorithm for pose detection and thanks to specific pose sequence, built an effective action recognition and classification system. Roshtkhari *et al.* [49] proposed coding of supervision video as a compact set of spatio-temporal volumes, which allows for action recognition without prior knowledge about actions. Rahman *et al.* [50] proposed to use the surrounding regions of the human silhouette termed as negative space for recognition of human actions from video sequences. In our recent work [51] the simple but dynamic geometric features like height, width, head and feet positions of the silhouette, combined with a finite state machine, were used for recognition of several human actions.

Activity recognition on the basis of analysis of a video stream can be performed in many ways and using a variety of digital image processing methods. Typically, the feature vector, allowing for the recognition, is calculated based on detected human silhouette. In [52] the feature vector consists of projections of silhouettes at the axes of the coordinate system, to which Fourier transform is then applied. In [53] simple parameters of human body (the length and width of the selected parts of the body, for example: lower and upper body part, head, *etc.*) are computed. The location of five significant body points (head, tips of the feet and tips of the hands) is based on skin color analysis and convex points of body shape [54].

## 3. General Overview of the System

**Figure 1.**
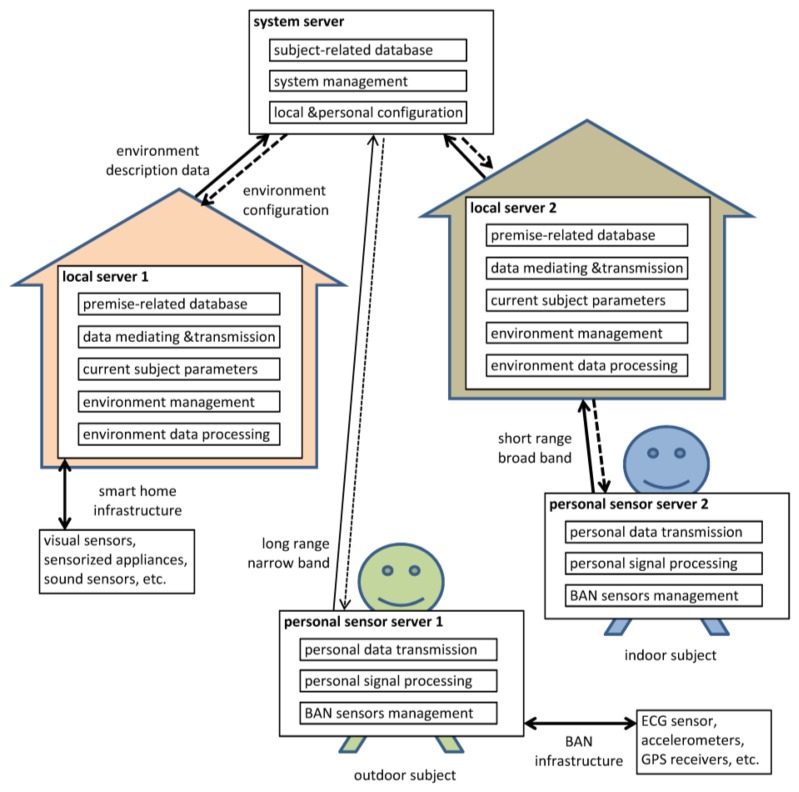
Backbone architecture of the surveillance system.

The proposed prototype design is based on an open architecture, allowing for *ad-hoc* modification of the sensor set and surveillance priorities. The architecture backbone (Figure 1) consists of a system server, (running the decision making algorithms, databases and system management) and multiple smart environments and personal sensor networks. Each smart environment corresponds to a subject's premise, uses specific infrastructure-embedded sensors and is managed by a local server. Each personal network corresponds to a particular subject, uses specific wearable sensors (*i.e*., selected with regard to a disease) and is managed by a wearable server. The personal sensor network may constitute an independent measurement node under control of the system server (in case when the subject is outdoors) or may be included in one of smart environments (e.g., in case when the subject is at home). In the latter case cooperation of the embedded and wearable measurement systems provides redundant information, used for optimization and calibration purposes. Although it is technically possible, we do not assume cooperation between the two personal networks at this stage.

The subject-dependent data are stored uniquely in the system server, and as a result, it can directly control personal servers. Moreover, identification of the subject in a particular environment implies uploading the respective personal data from the system server to the selected local server in order to enable the cooperation. This approach frees the supervised subject from any constraints associated with his or her particular environment and provides continuous monitoring, even for mobile subjects. Consequently, the general system architecture allows for the supervision of multiple subjects in multiple environments (homes, offices or vehicles), which was not considered in related papers.

Besides the remark above, the system architecture inherits features from other similar systems. Therefore in present paper we do not attempt to describe the whole system, but rather are going to highlight the main novel aspects resulting from an integrated approach.

## 4. From Detection to Decision

Making an unobtrusive measurement is a principle of measurements in all assisted living systems and thus it favors sensors embedded in the infrastructure of the subject's premise (apartment). On the other hand, the use of wearable sensors is the only option if seamless measurement has to be made on a mobile subject. A compromise aiming at optimal data reliability depends on several time-varying factors and thus has to be made continuously. In the proposed system we sought to benefit from the complementary features of wearable and premise-embedded sensors and to program their best possible cooperation rules.

Wearable sensors represent each particular subject, accompanying him or her in any activity in each environment he or she visits. This group of sensors is programmed for seamless data acquisition, commonly uses wireless data transmission and requires a power saving-oriented design. The wearable sensors are organized in a body area network (BAN) controlled and synchronized by a wearable server. The wearable server is also responsible for sensor-specific data processing (intelligent sensing) and communication with the system server. The wearable server is uniquely related to the subject, therefore all issued data bear the subject's identifier [55].

Sensors embedded in building infrastructures characterize a given environment and represent its changes caused by the actions of subjects. These sensors perform occasional recordings depending on subject presence, commonly use wired data transmission and do not make energy-related design issues. The embedded sensors are organized in a local area network (LAN) accepting *ad-hoc* connection of personal servers when subjects are present. In the case where multiple subjects share a common space, the system has to recognize particular subjects and apply an appropriate set of personalized behavior rules.

### 4.1. Management of Data and Hardware Resources

The technical design based on open architecture, allows for random configuration of active sensors by the software. Accordingly, the hardware configuration and data transfer protocol support seamless management of the sensor network in background of the performed measurement. The system design supports three levels of management:

- activating and deactivating of selected sensors,
- modification of setup in sensors' embedded software,
- appending or disconnecting a wearable network of a particular subject.

The decision making procedure is driven by the information on specific subjects' needs (e.g., heart or balance disorders), relations of the subject with the environment (e.g., connectability of the embedded and wearable sensor networks) and status of the sensor hardware (e.g., battery charge). Each configuration change is immediately reflected in the measurement data structure, allowing for correct interpretation of the sensors' outcome.

Technically speaking, the embedded sensors of the premise (intelligent cameras, microphone matrices, sensorized home appliances, *etc.*) and wearable sensors of the subject (positioning, acceleration and cardiac activity) are organized into two separate networks, each capable of independent data reporting. However, considering that the average subject spends a prevalence of time within the premise, subordination of his or her wearable network to the premise-embedded sensor network is beneficial at least for reducing the energy dissipation and broadening of transmitted data stream.

The architecture of the proposed monitoring system assumes that a smartphone (or mobile processing unit) plays the role of wearable server and integrates mobile sensors into a body area network (BAN), while the embedded server collects, processes and transmits premise-related data. The system server plays supervisory roles: it runs system databases, decision making software and the Internet interface for users, supervisors (e.g., doctors) and system administrator. The wearable part of the system was designed to monitor the state of health without constraining the activities of a wearer. This was achieved by using wireless data transmission modules and small, wearable, battery-operated sensors that can be attached to the body or hidden in the clothes.

The embedded server software is implemented in C# language and runs on a Windows 7 machine (Intel Quad Core Q8300 processor, 4 GB of RAM). The server is equipped with dual LAN, Bluetooth and WiFi network interfaces. It listens for incoming data on available network interfaces and forwards them to the database. Received data are analyzed by custom-built algorithms and forwarded to the system server, where medical data are accessible to the supervisor or other authorized people via an easy-to-use web-based graphical interface.

**Figure 2.**
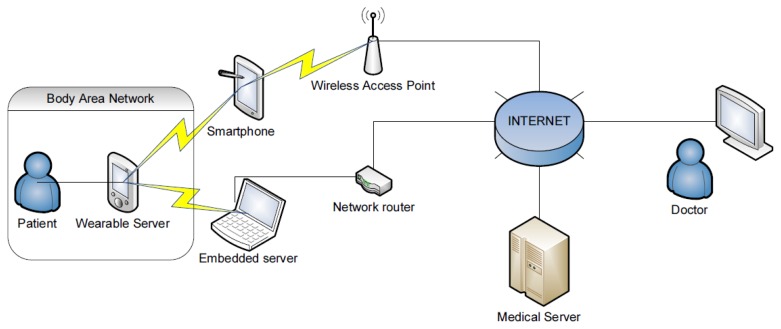
Human behavior tracing–network architecture.

The wearable server is based on an 8-bit 16 MHz ATmega microcontroller unit with a Cambridge Silicon Radio Bluetooth v.2.0 class 2 module. It is connected to four wearable sensors: dedicated ECG sensor (built based on Analog Devices medical amplifiers), temperature sensor, micromechanical Bosch BMA180 accelerometer and Pentagram GPS receiver (MTK chipset). Data from sensors (3-axis acceleration, GPS location, body temperature and ECG signal) are transmitted to the monitoring gateway via Bluetooth using designed data transmission protocol. The monitoring gateway, built with HTC Desire smartphone (119 × 60 × 11.9 mm), is used to acquire monitoring signal and forward it to the System Server. It is equipped with 1 GHz Scorpion CPU, GPS and has 576 MB RAM, what makes it very powerful processing unit. The smartphone runs Android OS v2.2 (Eclair). The network architecture is shown in Figure 2.

Data processing algorithms were implemented in C language for the main purposes of acquisition and processing of data from wearable sensors, and communication with the system server via an available wireless interface. Key functionalities of the software include calculating and displaying the heart rate (QRS detection algorithm), calculating the subject's speed based on GPS data and performing statistical analysis of acceleration signals in the selected time window. One of the most popular and often cited QRS detection algorithms that works in the time domain is the Pan and Tompkins algorithm proposed in 1985 [56]. The QRS detection algorithm is based on analysis of the slope, amplitude and width of the QRS complex which refers to the depolarization of the right and left ventricles. In order to reduce the noise, the ECG signal first passes through a digital bandpass filter composed of cascaded high-pass and low-pass filters. The next process after filtering is differentiation, followed by squaring, and then moving window integration.

For reasons of compatibility with a wide range of smartphone devices and operating systems, all other algorithms for the wearable server were implemented in Java. In order to locate the subject during the outdoor activities, tracking data are gathered by a GPS module and directly forwarded to the system server. The mobility record of the subject is accessible through web-based interface which uses Google Maps API in order to mark the subject's location on the map. The fall detection is performed by the algorithm analyzing data from the accelerometer. The absolute sum of tree-axis accelerometer data is calculated. If obtained value is higher than an experimentally set trigger point, the alarm module is switched on. The performance of wearable subsystem was investigated while performing complex outdoor activities. Results of testing for outdoor activities was shown in Section 5.2.

Combining the information from wearable and house-embedded sensors on a common timeline requires proper ordering and synchronization of recorded data packets. Two sources of delay were identified in the system:

– data processing delays, resulting from the usage of data buffers for averaging or analysis (e.g., Pan and Tompkins algorithm)–in consequence, the information about the event appears later than the event itself,
– data transmission delays, resulting from the usage of packetized data transmission (IP), integrity checking and possible retransmission of data blocks, in particular in wireless link–in consequence, the information about two events may be received in the wrong order.

Fortunately, data processing delays are constant and may be predicted based on the system design. Moreover, the implemented synchronization mechanism, based on the storage of sample acquisition times, enables one to calculate the delay accurately. During indoor activities the subject's action is represented by two data streams, and the correct alignment is achieved by searching for the best cross-correlation. Since the data originate from two different measurement methods (including physical background, processing and sampling frequency), the synchronization procedure is preceded by automatic selection of appropriate signal sections. For this purpose, we selected adaptable thresholds identifying the top 5% of the acceleration values in a history of specified duration (15 min) of either signal. If a section with locally maximal acceleration is found, the other signal history is screened again for occurrence of similar acceleration patterns. The correction of the previous delay time is made only when the cross-correlation value is significant (*i.e*., over 70%), thus not every behavior containing a rapid movement is used for synchronization.

Another issue revealed by the assumed flexibility of the system, is the format for machine representation of behavioral data. The solution commonly found elsewhere is a raw data timeline, and we also obtain data in such as format as the output of the synchronization procedure. This method is the most straightforward, but the lack of support for reconfigurable networks or sensor-side object recognition and the fast growing data volume are serious drawbacks from our point of view. The other option is based on storage of recognized behavioral items belonging to a predefined dictionary. This method, commonly applied in manned supervision procedures, is focused on recognition and verification of a given chain of actions without considering data reliability and assessment of possible danger. Finally, we selected a graph-based representation [57] using two levels of description: the outer for localization of the subject in the premise and the inner for description of his or her status (action and health). Due to the limited reliability of status recognition, the graph nodes at the inner level contain probability-ordered lists of detected status. One of the principal advantages of the graph-based descriptions of the behavioral data is the support for prediction of possible future actions and displacements. Irregular timelines of such graphs containing occasional links to raw data strips are stored separately in the system server as a behavioral record for each supervised subject.

### 4.2. Detecting of the Presence and Pose

Detection of the subject's presence in a particular room of the premise was based on image analysis. Accordingly to the guidelines for privacy protection and to legal regulations in several countries, no visual information can be transferred out of private premises. Therefore embedding the image analysis algorithms in the camera hardware is envisaged at the commercialization stage, although for experimental purposes we used a regular PC connected directly to a digital camera or recorder and running Matlab environment for programming and testing the algorithms.

In this study we decided to create the feature vector based on optical flow (OF). It is a straightforward approach coming from the belief that the sum of the small movements of the whole body (and thus the individual parts of it) is a visual manifestation of the operation performed.

Optical flow can be calculated using different algorithms [58,59]. Faster algorithms generate less accurate motion fields, but still sufficient to detect moving objects' silhouettes, while the calculation time for more accurate algorithms is too long to apply them in practice. The results from our research described in [60,61] show that both well-known gradient methods—Horn-Schunck [62] and Lucas-Kanade [63]—can be successfully applied.

In the presented study the Horn-Schunck algorithm was applied, which uses the first order difference as a method of numerical differentiation. The disadvantage of this differentiation method is the sensitivity to noise, while the advantage is the use of only two frames of a sequence. The obtained computation speed was satisfactory: the optical flow of 640 × 480 size image was calculated in 0.1 s on the computer Intel Core i7 920, 2.66 GHz, operating under the Windows 7 x64 system.

Optical flow was calculated for two frames which numbers differ by 3. It means that the time interval between two frames, used to calculate the OF, was equal to 120 ms (because the time between consecutive frames is 40 ms for 25 frames per second). During such a long time interval a significant shift was observed, even for slowly moving objects. For *n* consecutive steps of the algorithm, the numbers of pairs of frames used to compute OF were *n* × 3 and *n* × 3 + 3.

Detection of moving objects was performed by binarization of optical flow modulus (|OF|) with a constant threshold. The threshold's value was chosen experimentally during the previous experiments described in [59] (indoor human activities) and [60] (traffic analysis). It should be noticed that segmentation using OF is effective even if the contrast between the person and the background is low. The only assumption is that the color of the clothes of the observed person should not be completely uniform–which is usually met.

**Figure 3.**
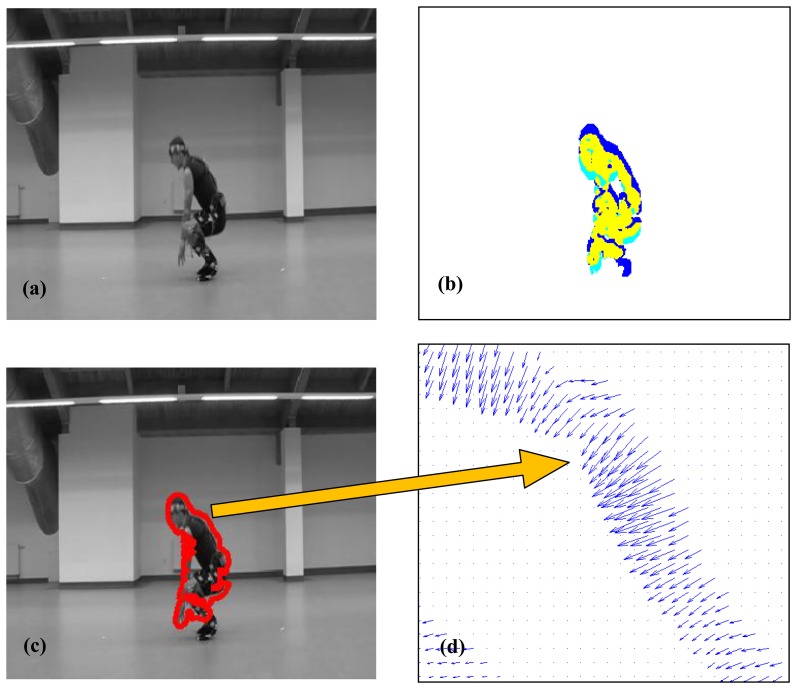
Optical Flow (OF) refinement: (**a**) Original image. (**b**) Two consecutive binarized OF superimposed. (**c**) Object edge. (**d**) Magnified OF masked by the dilated edge.

In the next step the common part of two consecutive binarized OFs was calculated thus enabling us to determine the actual shape of the silhouette (Figure 3b yellow part). The contour of the detected silhouette is shown in Figure 3c.

OF vectors located inside the silhouette outline have somewhat chaotic orientations. This comes from the simplicity of the optical flow method used. However, vectors located at the silhouette edge reflect quite well the direction of object motion (see Figure 3d) Therefore it was decided that the representation of motion was formed by vectors located on the silhouette edge extended (using morphological dilation) to about four pixels.

**Figure 4.**
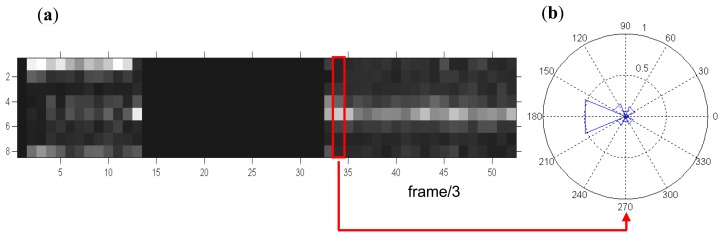
(**a**) Representation of the movie part by means of histograms of OF directions: motion to the right side of the scene corresponds to histogram bin equal to 1 (B1), motion to the left–bin equal to 5 (B5). Frames 1–13–a person is running to the right, 14–32–the scene contains only a static background, 33–52–a person is walking to the left, (**b**) Histogram of optical flow directions calculated within the dilated edge of the object (polar representation).

Figure 4a presents time-angle representations made from the analysis of a movie consisting of 160 frames. The vertical axis shows the histogram of the aggregated optical flow directions. Eight aggregated directions (bins: B1-B8) correspond to the following ranges of angles [−337.50 22.50], [22.50 67.50],…, [292.50 337.50]. Angles corresponding to the centers of those ranges are respectively: 0°, 45°,…, 315° (see also Figure 4b). On the horizontal axis the numbers of the analyzed frames divided by three are shown, because the movie was analyzed with three frame intervals. The higher value in a given histogram bin, the brighter corresponding field.

In Figure 4b an exemplary normalized histogram of the directions of optical flow vectors aggregated to eight compartments (bins: B1–B8) is shown in polar form. Histogram values corresponding to one feature vector are marked in Figure 4a.

Visual analysis of Figure 4a corresponds to the real activities registered on the exemplary film. In the first phase a person is running to the right side of the scene (range 1–13 on the horizontal axis). The direction to the right side corresponds to the first bin of the histogram, so this row is characterized by high values. In the range of 14–32 the scene is static – histogram values are equal to zero. In the range of 33–52 the investigated volunteer is marching fast to the left side. This situation is reflected on histograms with high values of the 5th bin. On the basis of vectors presented in Figure 4 the performed activity is recognized.

### 4.3. Complementary Measurements for Recognition of Activity

Striving for a minimally-intrusive set of universal wearable sensors, we found it reasonable to track the subject's position, heart rate and mobility. More specific sensors (e.g., glucometer for diabetics, or oxymeter for apnea-endangered) may be applied at any time and immediately integrated with the wearable sensor network. The details on positioning and cardiac data are beyond the scope of this paper, however it is worth mentioning that accelerometer data, besides representing the subject's mobility, are also used in qualification of cardiac rhythm changes.

For engineering a surveillance system for elderly, monitoring their activity with the conditional use of intelligent cameras as premise-embedded sensors and accelerometers as wearable sensors of motion, it is interesting how far the information gathered both ways is coherent. Signal strips from wearable accelerometers are attributed with a unique timestamp by the wearable server, which was designed to provide an accurate time counting mechanism. It solves the difficulty of synchronization between the video and the wireless accelerometers.

For these purposes the experiments with simultaneous acquisition of video silhouette motion and sternum acceleration trajectory of 20 volunteers (eight women and 12 men, age between 22 ÷ 61 years) were precisely prepared and carried out in the area of large gym in AGH-UST swimming pool. Time synchronization of the sensors has been realized by the registration of a movement, whereby characteristics of signals from various sensors clearly indicate its beginning or end. Therefore, the selected movement was a free jump with both feet up. The synchronization point was taken as the first moment of contact of the front foot (toes) of a subject with the ground after the jump. The synchronization of outdoor measurements from wearable accelerometers is based on timestamp mechanism performed by the on-board microcontroller. The time of acquisition of each signal strip is stored for synchronization purposes.

Simultaneous recording of selected events by video sensor and by a wearable wireless accelerometer as well as comparison of the timestamps mechanism allows for assessment and compensation of time delay resulted from different data processing and transmission. The maximum acceleration-based synchronization was then adopted for automatic compensation of delay of data packets recorded from wearable and house-embedded sensors (see Section 2.1). Each volunteer was asked to perform about 30 repetitions (Table 1) of 12 physical activities most common in daily living at home (Table 2).

**Figure 5.**
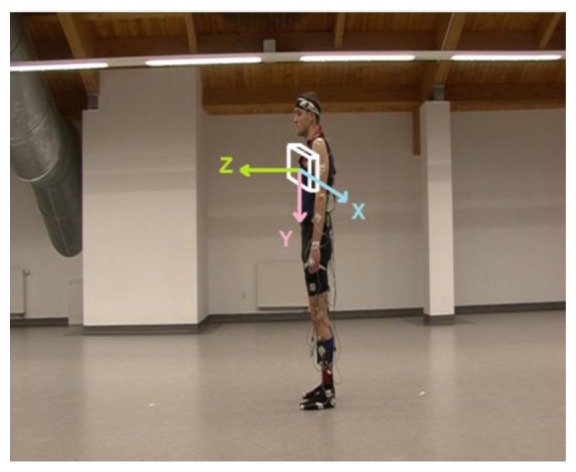
Video frame presenting a view from the camera. White edge box shows a placement and 3-axis orientation of the accelerometer.

Silhouette motion was registered by means of digital video camera (Sony HDR-FX7E) fixed at a constant height and a constant distance from the examined subject. As presented in Figure 5, the camera was situated on the left side of the human body since this alignment emphasized subject's motion registration in a sagittal plane. The sampling rate of 25 frames per second and the frame size of 720 × 576 pixels (according to the DV-PAL format) were used.

The online measurements (via Bluetooth) of 3-axis acceleration with the sampling frequency of 100 Hz were performed with the use of Revitus module [64]. The location of the accelerometer on the sternum was used to obtain the signal which best characterized and distinguished the examined physical movements. The 3-axis orientation of the accelerometer is illustrated in Figure 5.

After the experimental stage, a dedicated preprocessing was applied to data collected from the digital camera and accelerometer sensors. The human activities specified in Table 2 were classified in temporal window which length was selected experimentally to 160 s. This selection was performed by means of cumulative histograms of time durations calculated for all volunteers and for motion activities of all examined types.

**Figure 6.**
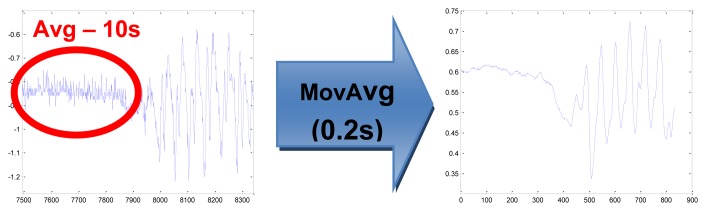
Preprocessing of the acceleration signals–subtracting the local offset value and averaging the signal.

Finally, the feature vector built of optical flow-based motion directions histogram bins B1, B2, …, B8 was prepared as follows: [B1 B2 B3 B4 B5 B6 B7 B8]. Each of the bins corresponds to one of eight directions described in Section 4.2 [65]. The successive preprocessing steps for the acceleration signals are presented below [65]:

- subtracting the local offset value (calculated as average signal value of 10 s signal period in a motionless stand pose), separately for each axis (*x*,*y*,*z*) and for each person (Figure 6)
- averaging the signal in 0.2 s time window in order to eliminate distortions and to extract the main direction of the signal changes (Figure 6)
- normalizing of amplitude separately for each person – dividing the signal value by the maximal absolute value of all the measurements of all the activities for the person
- composing the feature vector consisting of the processed acceleration signals in 3-axis as follows: [x y z]
- normalizing of feature vector amplitudes into the range of (0,1]
- fourfold subsampling from the sampling frequency of 100 Hz to 25 Hz.

A feature vector for combination of video and acceleration sensors contained the feature vectors for each of the sensors: [B1 B2 B3 B4 B5 B6 B7 B8 X Y Z].

For recognition purposes of the selected motion activities the supervised *k*-Nearest Neighbors (*k*-NN) classification was applied [66–68]. By means of a Leave-One-Out (LOO) method, the value of *k* parameter was determined to be 1. The data set of all measurements was divided into two parts–learning and testing sets. In the learning set there were 2,400 randomly selected activities (10 from each of 12 types and from each of 20 people). The testing set consisted of remaining 4,874 activities, not drawn from the learning set.

Results of these experiments are helpful for human-unattended selection of the best sensor in several typical circumstances and to assess the reliability of the worst sensor. The reliability coefficient helps validating the accelerometer data in case when the subject is not in the video detector range (e.g., outdoors).

### 4.4. Learning of Behavior and Detection of Danger

The behavioral record resulting from the ongoing measurements taken on the subject is processed in the system server by the danger detection mechanism based on two databases:

- Premise-related database describing the purpose and topological connections between pieces in the subject's premise,
- Subject-related database describing usual behavioral patterns and their variants for each part of the day [69].

The premise-related database specifies the intended usage of each piece in the premise and the rules of operating of sensorized appliances. It contains general limitations on poses at specific locations (e.g., no lying is allowed in the kitchen) and equipment (e.g., the electric kettle may be removed from its base for no longer than 90 s). The layout of the premise is also specified here in a form of probability of subject's displacement from one zone to another. The term “zone” is used as equivalent of “piece”, however several zones may be defined in one piece in case of differences of their expected usage.

The behavioral pattern used for description of action specificity of each subject (e.g., habits) is a statistically processed section of behavioral record including: label of current status, average and standard deviation values of its expected duration, probability-ordered list of subsequent status and optional pointers to selected examples of detailed subject-related sensor data.

Calculating the statistics of behavioral patterns and database search for similar past behavior is based on calculation of the distance metric and needs a definition appropriate for the domain of behavior descriptors. Several distance measures were studied in [70] with the use of real behavior measurement data. Finally, the dynamic time-warping (DTW) algorithm [71,72] was selected. Since our definition of pose is based on both visual and accelerometer data, we combined the approaches proposed by Rahman *et al.* [50] and Liu *et al.* [44].

For two behavioral patterns, *x*_1_ and *x*_2_, being sequences of human actions of different length *l*_1_ and *l*_2_ respectively, DTW calculates the optimal alignment of their descriptive sequences. At a given time, any subject state is considered as a linear combination of *P* = 7 (see Table 2 + “undetermined”) elementary poses and represented by their contribution coefficients *w_p_*. Although the poses are not purely orthogonal, we calculate the distance between states as Euclidean distance in 7-dimensional space. The algorithm starts with constructing an *l*_1_-by-*l*_2_ distance matrix *d* such that:

(1) $$d[i,j]=\sum_{p=1}^{P}{\left(w_{p,1}[i]-w_{p,2}[j]\right)}^{2}$$

Each value in this matrix indexed by (*i*,*j*) represents the square of the difference between subject states *x*_1_[*i*] and *x*_2_[*j*] at different time points *i* and *j*. A particular alignment corresponds to a path *φ* through the distance matrix of the form:

(2) $$\begin{array}{cc} \varphi (k)=\left(\varphi_{1}(k),\varphi_{2}(k)\right), & 1\leq k\leq K \end{array}$$

where *φ*_1_ and *φ*_2_ represent row and column indices into the distance matrix, and *K* is the alignment length. The DTW method yields the optimal alignment that minimizes the overall cost:

(3) $$C(x_{1},x_{2})=\underset{\varphi }{\min}C_{\varphi }(x_{1},x_{2})$$

where C*_φ_* is the total cost of the alignment path *φ* and is defined as:

(4) $$C_{\varphi }(x_{1},x_{2})=\sum_{k=1}^{K}d\left[x_{1}[\varphi_{1}(k)],x_{2}[\varphi_{2}(k)]\right]$$

and *x*_n_ are subject states represented by values *w_p_*_,_ *_n_* being contributions of elementary poses *p* in the state *x*.

The final energy difference between the two behavioral patterns *x*_1_ and *x*_2_, is given by the cost of their optimal alignment, and depends on both the value differences between the two patterns, as well as the length *K* of the alignment (which reveals the local temporal difference between two patterns). The DTW quantifies changes in morphology resulting from state-space amplitude and timing differences between two patterns. Using this information, behavioral patterns carrying information of possible danger are clustered in separate groups, with appropriate unique symbols.

**Figure 7.**
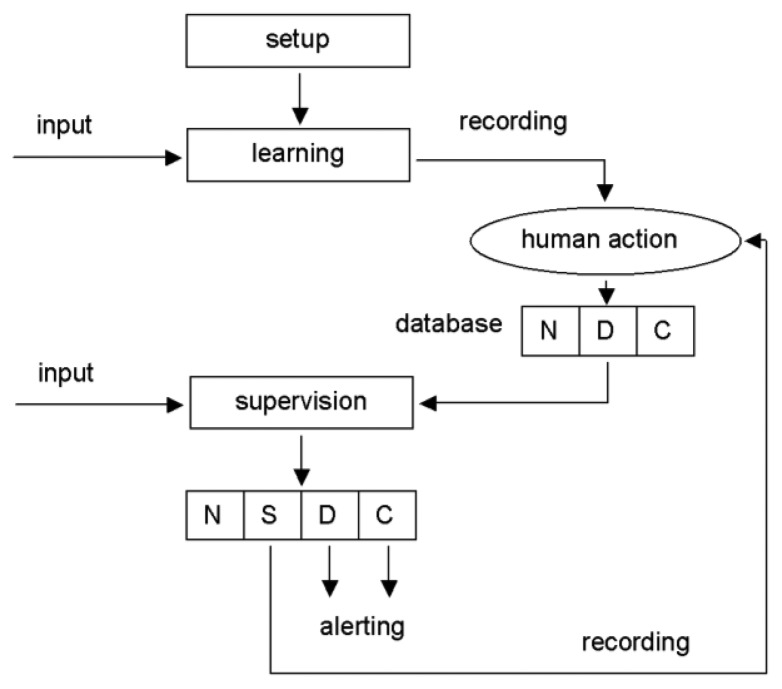
Block diagram of the life cycle of the assisted living system. Abbreviations “N”, “S”, “D” and “C” stand for following categories of subject behavior: normal, suspicious, dangerous and critical.

Before being applied for the danger detection mechanism, the contents of both databases have to be initialized. Although basic information on expected subject behavior in particular zones is entered manually, behavioral patterns are entered as examples recorded by the same system that is used for surveillance for preserving the specificity of subjects' actions. Unfortunately, intentional recording of patterns for a variety of possible dangerous or critical behaviors by the subject is not feasible. Therefore we initially perform recordings of patterns of all behavior available in everyday life and then, in a series of interview with a human operator, selected patterns are indicated as examples of dangerous behavior. During the later operation of the surveillance system, all recordings that not match the patterns of safe behavior are recorded as suspicious (together with respective raw data streams) and subject to a visual inspection by the human operator. The danger detection mechanism runs either in setup, learning and discrimination modes (Figure 7):

- In the setup mode, the human operator (supervisor) defines the premise-specific permission list, redundant sensors and sensor overlapping areas and configures the alerting rules.
- In the learning mode the system records the subject's behavior and calculates statistics of behavioral patterns. In case the permission list or subject-related limits are exceeded, or in case the subject presses the button, detailed data from selected sensors are recorded for future review.
- In the supervision mode the system records the subject's behavior, calculates statistics of behavioral patterns and performs alerting as programmed. For all events classified as ‘suspicious’ the detailed data from selected sensors are recorded for further review.

## 5. Results and Discussion

### 5.1. Results of Testing for Indoor Activities

The main components of the system were tested under laboratory conditions with the help of volunteers playing out different test poses and scenarios. Tests with elderly volunteers in a dedicated apartment-like laboratory are currently being prepared and tests in domiciles of elderly volunteers are scheduled for a three-year project.

The concept of complementary use of sensors from embedded and wearable networks for improvement of data reliability was verified in a series of elementary activities detected and classified based on single sensors and multiple sensor signals. Results of tests for separate visual and accelerometer-based detection and for combined modes detection are summarized in the following tables.

Table 3 presents the percentage of correctly recognized activities for individual sensors and for their combination. See Table 2 for coding of types of physical activities. The last column of the table contains values of the recognition correctness when all of the examined types of activities were taken into account.

Table 4 contains the percentage of recognition correctness of all examined physical activities for each of the 20 volunteers.

Based on the presented summary of the final results it can be concluded that the combination of two sensors enables to improve reliability of the activities recognition. Some type of the activities (6a,6b,3b,4b) were less reliably recognized than others. Probably worse gait results (6a,6b) can be attributed for too high diversity of walking rhythm for different volunteers. Reaching activities were also difficult to recognize, since there are characterized by low level of whole human body dynamics.

### 5.2. Results of Testing for Outdoor Activities

Separate testing was performed for monitoring of outdoor activities captured by a unique use of wearable sensor network controlled and synchronized by the wearable server.

The experiment setup for outdoor activities consisted of wearable server, Bosch BMA180 accelerometer and GPS receiver (MTK chipset). However, system monitoring capability can be extended by adding up to two more sensors in an *ad-hoc* mode. Our other studies [73,74] proved the possibility of integrating the ECG sensor, the temperature sensor and the skin humidity sensor into this system.

The aim of the experiment was to investigate the system performance during complex outdoor activities and the ability of recognition of selected subject states while performing different activities. The experiment was carried out in an open area (garden). All sensors were attached to subject's body or hidden in the clothes. A connection between wearable server and system server was established via a wireless 3G network. The implemented software controlled data transmissions during the experiment.

**Figure 8.**
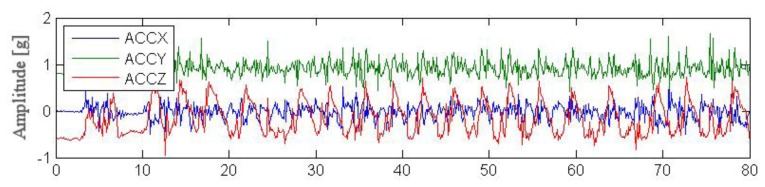
Data acquired from wearable accelerometer in 80s time window during digging a hole.

**Figure 9.**
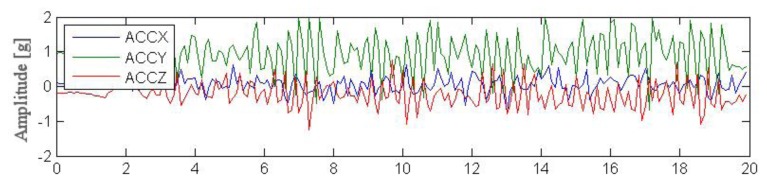
Data acquired from wearable accelerometer during a 20 s run.

**Figure 10.**
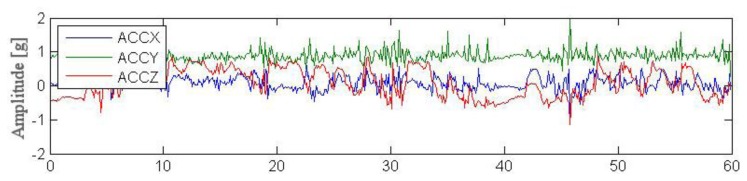
Data acquired from wearable accelerometer 60 s trees trimming.

Outdoor testing was carried out by four young volunteers performing simple actions such as standing, walking, running and next performing complex activities in the garden. Each session lasted for ca. 2 min and aimed to simulate an excerpt of everyday life. Figures 8, 9 and 10 illustrate excerpts from signals of acceleration changes (3D accelerometer data [ACCX, ACCY, ACCZ]) during different complex garden activities including digging a hole, running, trimming trees. Tables 5, 6 and 7 show results of basic statistical analysis of acquired signals (MEAN–mean value, STD–standard deviation, MIN-minimal value, MAX-maximal value).

Our measurements show that it is possible to establish an open architecture wearable wireless sensor network and apply it to monitor complex outdoor activities of humans. Furthermore, the implemented algorithms allowed for calculation of a two-dimensional feature vector which consists of the average of the normalized sum of accelerometer data and subject speed from the GPS sensor. Based on this feature vector we achieved an overall accuracy of 80% in recognition of different activities. This study demonstrated the possibility of applying wearable subject-specific sensors for human behavior tracing under the specified outdoor conditions.

## 6. Conclusions

A system for seamless tracing of human behavior using complementary wearable and house-embedded sensors was designed according to a three-layer architecture allowing for separate adaptation of sensors (in response to particular medical needs) network organization (in response to subjects or premise changes) and behavioral record interpretation (in response to personal habits). The system automatically collects the data from the best available sensors and performs energy-aware selection of data gateways in order to increase the operation time of wireless body area sensor networks. The system automatically compensates the processing and transmission delay based on two acceleration-representative data series, and evaluates the reliability of wearable accelerometers-based outdoor measurements of mobility with the use of cross matching of acceleration patterns recorded during indoor tracing.

Putting aside possible delay and inaccuracy of values of some status components, the subject's behavior may be captured and analyzed in real time (total delay below 2 s) in order to detect abnormalities and to warn about possible health dangers. Such preventive analysis is very welcome for general surveillance of elderly people living on their own, as well as in specific circumstances such as car driving or similar activities.

The experiments performed by means of different sensors enabled us to establish a range of interchangeability and to choose the most appropriate sensor or sensor set for the specific application. Results of the research clearly confirmed that the analysis of human motion recorded with different methods leads to convergent conclusions, which can be used in home care monitoring or assisted living. It is possible to identify the selected movements in human daily life with a significant probability using single video or acceleration sensor and we demonstrated how the recognition accuracy raises with simultaneously using of both sensors.

Although our prototype does not address all the issues mentioned in the ‘Introduction’, we successfully implemented the prototype system for seamless tracing of human behavior using complementary wearable and house-embedded sensors. Our system uses an open architecture and allows for monitoring of multiple subjects in multiple smart environments (homes, offices or vehicles). Thanks to the double communication gateway of personal server, the subject stays connected to the system without limitation on his or her mobility. Particular sensors may be remotely switched on and off accordingly to the individual needs of the subject. The system learns from the subject's actions manually classified as regular, detects any exemption on behavior in real time, alerts and records corresponding data for further review.

For an indoor subjects, the activity recognition is based on synergy of accelerometer data gathered by a wearable sensor network, and results of optical flow-based visual data analysis. In case of outdoor subjects, the system has to rely on accelerometers only, and thus the recognition accuracy is lower. Our experiments show that the visual method is more reliable than the accelerometer-based method. Short activity sequences recorded simultaneously by both systems is used to establish a consensus on subject's behavior, but also to measure differences between methods, which help to assess the reliability of accelerometer-based method when the subject goes outdoor.

This paper presents selected issues we solved during designing a complex surveillance system using relatively simple sensors. It was shown that it is possible to acquire data from wearable sensors during performing complex activities in outdoor conditions. At the current stage of the prototype, there are several limitations of the system including:

– temporary usage of image processing-dedicated computer instead of intelligent camera
– short operating time of wearable server (only *ca*. 5 h)
– risk of system damage during the fall of the subject or
– possible interferences with radio operating devices during wireless data transmission.

These limitations will be successively reduced by using embedded algorithms for analysis of video and vital signs, reducing the periods of wireless transmission activity and future works with smart fabrics allowing for prototyping a measurement clothes for most pertinent vital signs.

Principal applications of the system include early detection of abnormal conditions and remotely supervised rehabilitation. However, it can also provide the medical community with an opportunity to investigate patients at their homes, for personalized trends and group patterns, allowing insights into disease evolution, the rehabilitation process, and the effects of drug therapy. The technical merit of the system is only the beginning of its eventual success. In fact, the acceptance of the system by the elderly is a key issue. We will start the real-world implementation of the system using high-risk subjects (e.g., prone to languor) and accepting some degree of inaccuracy. At the next stage, the decision making software will be revised and we plan to have 200 copies of the personal subsystem manufactured and implement it in private homes, a nursing home and a psychiatric hospital. These implementations are expected to return statistically relevant results on the social and medical advantages of the system and on its usability.

## Figures and Tables

**Table 1.** A number of repetitions of each movement for each activity and for each volunteer (V1 ÷ V20).

	**1a**	**1b**	**2a**	**2b**	**3a**	**3b**	**4a**	**4b**	**5a**	**5b**	**6a**	**6b**
V1	30	30	28	28	31	31	36	36	30	30	19	19
V2	30	30	31	31	30	30	30	30	30	30	25	25
V3	30	30	32	32	30	30	30	30	30	30	31	31
V4	28	28	31	31	30	30	46	46	40	40	28	28
V5	29	29	30	30	30	30	31	31	30	30	27	27
V6	31	31	30	30	33	33	30	30	30	30	21	21
V7	29	29	30	30	31	31	30	30	31	31	22	22
V8	27	27	30	30	29	29	28	28	30	30	23	23
V9	30	30	30	30	31	31	30	30	31	31	24	24
V10	29	29	32	32	30	30	33	33	33	33	28	28
V11	30	30	29	29	30	30	31	31	37	37	29	29
V12	30	30	33	33	30	30	29	29	30	30	29	29
V13	30	30	30	30	32	32	31	31	37	37	29	29
V14	30	30	30	30	30	30	30	30	41	41	35	35
V15	30	30	30	30	30	30	30	30	30	30	32	32
V16	29	29	29	29	30	30	30	30	29	29	30	30
V17	30	30	21	21	32	32	30	30	30	30	28	28
V18	30	30	30	30	31	31	30	30	30	30	40	40
V19	30	30	30	30	30	30	30	30	30	30	40	40
V20	30	30	30	30	30	30	30	30	30	30	35	35

**Table 2.** Examined physical activities.

**Activity symbol**	**Activity description**

**1a**	Going from stand to squat pose
**1b**	Going from squat to stand pose
**2a**	Sitting on the chair from stand pose
**2b**	Standing up from the chair to stand pose
**3a**	Reaching forward with the left upper limb in a sagittal plane (in stand pose)
**3b**	Return from reaching forward with the left upper limb in a sagittal plane (in stand pose)
**4a**	Reaching upward with the left upper limb in a sagittal plane (in stand pose)
**4b**	Return from reaching upward with the left upper limb in a sagittal plane (in stand pose)
**5a**	Bending forward the trunk from stand pose (in a sagittal plane)
**5b**	Straightening the trunk from bend to stand pose (in a sagittal plane)
**6a**	Single step for the right lower limb (support phase for the right lower limb)
**6b**	Single step for the left lower limb (support chase for the left lower limb)

**Table 3.** Recognition correctness of examined physical activities (1a ÷ 6b).

	**1a**	**1b**	**2a**	**2b**	**3a**	**3b**	**4a**	**4b**	**5a**	**5b**	**6a**	**6b**	**1a÷6b**

**VIDEO**	99.7	99.5	95.5	95.5	99.3	97.6	96.0	79.8	99.3	99.3	91.7	92.3	95.5
**ACC**	95.2	97.2	95.5	94.2	96.6	95.1	98.4	97.6	97.9	99.3	96.5	96.0	96.7
**VIDEO + ACC**	99.7	100.0	99.7	100.0	99.8	98.0	99.1	96.2	99.5	99.8	97.6	97.1	98.9

**Table 4.** Recognition correctness of all examined physical activities for each of the volunteers (V1 ÷ V20).

	**VIDEO**	**ACC**	**VIDEO+ACC**

**V1**	97.4	96.9	99.1
**V2**	95.3	93.1	96.1
**V3**	95.9	99.2	99.2
**V4**	88.8	96.9	98.6
**V5**	97.9	98.3	99.1
**V6**	99.1	98.7	100.0
**V7**	99.6	100.0	100.0
**V8**	97.7	76.2	99.1
**V9**	94.4	93.1	98.7
**V10**	93.6	97.6	95.6
**V11**	99.2	99.2	100.0
**V12**	81.0	99.2	99.2
**V13**	96.5	91.1	98.8
**V14**	94.9	98.2	98.5
**V15**	99.2	100.0	99.6
**V16**	98.3	98.3	99.6
**V17**	92.3	94.6	99.5
**V18**	97.3	100.0	100.0
**V19**	97.7	100.0	100.0
**V20**	94.8	100.0	97.2

**Table 5.** Statistical analysis of experimental data acquired during 80 s digging a hole.

**Parameter**	**MEAN**	**STD**	**MIN**	**MAX**

ACCX [g]	−0.02	0.16	−0.51	0.60
ACCY [g]	0.91	0.17	0.42	1.66
ACCZ [g]	−0.16	0.35	−0.95	0.73

**Table 6.** Statistical analysis of experimental data acquired during a 20 s run.

**Parameter**	**MEAN**	**STD**	**MIN**	**MAX**

ACCX [g]	0.06	0.22	−0.73	0.63
ACCY [g]	0.93	0.55	−0.68	2.00
ACCZ [g]	−0.23	0.37	−1.24	0.77

**Table 7.** Statistical analysis of experimental data acquired during 60 s trees trimming.

**Parameter**	**MEAN**	**STD**	**MIN**	**MAX**

ACCX [g]	0.09	0.20	−0.95	0.59
ACCY [g]	0.88	0.17	0.27	1.99
ACCZ [g]	0.11	0.43	−1.13	1.02
